# Antimicrobial resistance: translating political commitment into national action

**DOI:** 10.2471/BLT.17.191890

**Published:** 2017-04-01

**Authors:** Hajime Inoue, Ren Minghui

**Affiliations:** a Office of the Director-General, World Health Organization, Avenue Appia 20, 1211 Geneva 27, Switzerland.; b Cluster for HIV/AIDS, Tuberculosis, Malaria and Neglected Tropical Diseases, World Health Organization, Geneva, Switzerland.

Antimicrobial resistance is one of the most complex global health challenges today. The world has long ignored warnings that some antibiotics are losing effectiveness after decades of overuse and misuse in human medicine, animal health and agriculture. Antivirals, antiparasitic agents and antifungals are also becoming increasingly ineffective. Though we live in an age of technology and innovation, we could soon find ourselves in an era where simple infections kill millions of people every year.

Common illnesses like bacterial pneumonia, post-operative infections, certain cancers, as well as the world’s biggest infectious killers – tuberculosis, human immunodeficiency virus (HIV) and malaria – are increasingly difficult to treat because of the emergence and spread of drug resistance.^1^ Worsening antimicrobial resistance could have serious public health, economic and social implications around the world. The World Bank has warned that antimicrobial resistance could cause as much damage to the global economy as the 2008 financial crisis.^2^

Antimicrobial resistance can only be tackled through a concerted global effort, led by heads of state and global institutions, and through coordinated action by the health and agricultural sectors, in partnership with the food industry, campaign groups and community organizations. Governments need closely aligned policies on the responsible use of medicines in human and animal health, and new standards for antibiotic use in agriculture and food production. A one health approach, involving close coordination among all relevant sectors and actors, should be urgently implemented by all governments.

In the past two years, we have seen global political momentum for addressing antimicrobial resistance. In May 2015, at the Sixty-eighth World Health Assembly, governments adopted a global action plan which identifies a set of strategic objectives.^1^^,^^3^ In 2016, the United Nations General Assembly held the first high-level meeting on antimicrobial resistance and passed a political declaration.^4^ The issue has also been on the agenda of recent group of seven (G7) and group of twenty (G20) meetings.

Since May 2015, progress has also been made in the implementation of these global commitments. Over one hundred countries have completed, or are about to complete, their national multisectoral action plans. WHO has established a global antimicrobial resistance surveillance system to track which drug-resistant pathogens are posing the biggest challenge.^5^ In May 2016, the Drugs for Neglected Diseases initiative and WHO launched a global research and development partnership to develop new antibiotics and promote their responsible use.^6^ In August 2016, WHO updated its guidelines for the prevention and treatment of the three common sexually transmitted infections– chlamydia, gonorrhoea and syphilis.^7^ Based on a review and analysis of national guidelines and prescribing practices for 20 common syndromes, WHO is revising the antibiotics included in the WHO model list of essential medicines.^8^ The organization has also rolled out a global awareness-raising campaign targeting policy-makers, health and agriculture workers and communities.

To scale up activities, governments can build on existing regulatory frameworks, surveillance systems, laboratory and infection control infrastructure and human resources that are already in place to manage drug resistance in tuberculosis, HIV and malaria. Diagnostic tools, logistics and technologies for sharing data can be used to link programmes at the country level. Most supranational tuberculosis reference laboratories have already confirmed they could expand susceptibility testing for other pathogens, should funding be available.

Work on drug-resistant tuberculosis, HIV and malaria also requires an accelerated effort and substantial investments in research and development. Multidrug-resistant tuberculosis killed an estimated 250,000 people in 2015 and – as an airborne disease – is considered a global health security risk.^9^ For viruses, for example, increasing HIV drug resistance is complicating efforts to expand access to effective treatment, threatening the global goal of ending the epidemic by 2030. For parasites, the spread of drug-resistant malaria is also a major concern.

Both at global and country level, there is still much more that needs to be done. An ad hoc interagency coordination group is being established by the United Nations (UN) Secretary-General, in consultation with WHO, the Food and Agriculture Organization of the UN and the World Organisation for Animal Health. WHO is preparing proposals for a global development and stewardship framework to support the development, control, distribution and appropriate use of new antimicrobial medicines, diagnostic tools, vaccines and other interventions. By May 2017, all countries should have their national action plans ready, as called for by World Health Assembly resolution 68.7.^3^

To see tangible progress, these global commitments must be translated into coherent regional and national action across the entire spectrum of diseases and pathogens.
